# Draft Genome Sequence of *Pseudoalteromonas tetraodonis* Strain UCD-SED8 (Phylum *Gammaproteobacteria*)

**DOI:** 10.1128/genomeA.01276-15

**Published:** 2015-11-05

**Authors:** Ruth D. Lee, Guillaume Jospin, Jenna M. Lang, Jonathan A. Eisen, David A. Coil

**Affiliations:** a Davis Genome Center, University of California, Davis, California, USA; b Department of Evolution and Ecology and Department of Medical Microbiology and Immunology, University of California, Davis, California, USA

## Abstract

Here, we present the draft genome sequence of *Pseudoalteromonas tetraodonis* UCD-SED8, a marine bacterium normally associated with the production of tetrodotoxin in pufferfish. This strain was isolated from sediment samples surrounding *Zostera marina* roots collected from Bodega Marine, California. The assembly consists of 4,017,727 bp contained in 35 contigs.

## GENOME ANNOUNCEMENT

*Pseudoalteromonas tetraodonis* UCD-SED8 was isolated from sediment samples surrounding common eelgrass (*Zostera marina*) roots grown at Bodega Marine Laboratory (Bodega, Bay, California, USA). The eelgrass sampling site was located north of Westshore Park, California, USA (38°19′10.0″N, 123°03′13.8″W). *P. tetraodonis* is a deep-sea marine bacterium that normally produces the neurotoxin tetrodotoxin, in association with pufferfish (*Fugu poecilonotus*) (1). It was originally described as *Alteromonas tetraodonis* (2); recent phylogenetic analysis, however, has confirmed its taxonomic association with the phylum *Gammaproteobacteria*. As a result, its identity was emended to *Pseudoalteromonas tetraodonis* (1).

Serial dilutions of sediment of 1:100 and 1:1,000 were made and spread on an altered seawater nutrient agar medium (ATCC Medium 2205, using InstantOcean in place of synthetic seawater), grown at room temperature for 24 h, and individual colonies were dilution-streaked onto seawater nutrient agar petri plates. DNA was extracted from a fresh 5-mL overnight culture using a Wizard Genomic DNA purification kit (Promega). Sanger sequencing was performed on the PCR-amplified 16S rRNA product (27F and 1391R PCR primers). BLAST and phylogenetic analyses (3) were used to identify this isolate.

A Nextera DNA sample prep kit (Illumina) was used to make a paired-end library (Illumina). Libraries were sequenced on an Illumina MiSeq platform, at a read length of 300 bp. A total of 3,029,779 high-quality paired-end reads were processed by the A5 assembly pipeline (4). This pipeline automates error correction, data cleaning, contig assembly, scaffolding, and quality control. The resulting assembly consisted of 35 contigs (longest: 921,029 bp; *N*_50_: 499,738) that were submitted to GenBank. This final assembly had 4,017,727 bp with a GC content of 40.0% and an overall coverage estimate of ~380×. Genome completeness was assessed using the PhyloSift software (5), which searches for a list of 37 highly conserved, single-copy marker genes (6), of which all 37 were found in this assembly.

The RAST server was used to perform an automated annotation (7). *P. tetraodonis* UCD-SED8 contains 3,627 predicted protein-coding sequences and 118 predicted noncoding RNAs.

### Nucleotide sequence accession numbers.

This whole-genome shotgun project has been deposited at DDBJ/EMBL/GenBank under the accession number LITK00000000. The version described in this paper is the first version, LITK01000000.
